# Assessing radiation-induced carotid vasculopathy using ultrasound after unilateral irradiation: a cross-sectional study

**DOI:** 10.1186/s13014-022-02101-7

**Published:** 2022-07-23

**Authors:** Judith T. Pruijssen, Joyce Wilbers, Frederick J. A. Meijer, Sjoert A. H. Pegge, Jacqueline J. Loonen, Chris L. de Korte, Johannes H. A. M. Kaanders, Hendrik H. G. Hansen

**Affiliations:** 1 Department of Medical Imaging/Radiology, Medical Ultrasound Imaging Center (MUSIC), Radboud University Medical Center, Nijmegen, The Netherlands; 2 Center of Expertise for Cancer Survivorship, Radboud University Medical Center, Nijmegen, The Netherlands; 3 Department of Imaging/Radiology, Radboud University Medical Center, Nijmegen, The Netherlands; 4 Physics of Fluids Group, TechMed Center, University Twente, Twente, The Netherlands; 5 Department of Radiation Oncology, Radboud University Medical Center, Nijmegen, The Netherlands

**Keywords:** Carotid vasculopathy, Radiotherapy, Intima-media thickness, Shear wave elastography, Pulse wave velocity

## Abstract

**Background:**

Increased head and neck cancer (HNC) survival requires attention to long-term treatment sequelae. Irradiated HNC survivors have a higher ischemic stroke risk. However, the pathophysiology of radiation-induced vasculopathy is unclear. Arterial stiffness could be a biomarker. This study examined alterations in intima-media thickness (IMT) and stiffness-related parameters, shear wave (SWV) and pulse wave velocity (PWV), in irradiated compared to control carotids in unilateral irradiated patients.

**Methods:**

Twenty-six patients, median 40.5 years, 5–15 years after unilateral irradiation for head and neck neoplasms underwent a bilateral carotid ultrasound using an Aixplorer system with SL18-5 and SL10-2 probes. IMT, SWV, and PWV were assessed in the proximal, mid, and distal common (CCA) and internal carotid artery (ICA). Plaques were characterized with magnetic resonance imaging. Measurements were compared between irradiated and control sides, and radiation dose effects were explored.

**Results:**

CCA-IMT was higher in irradiated than control carotids (0.54 [0.50–0.61] vs. 0.50 [0.44–0.54] mm, p = 0.001). For stiffness, only anterior mid-CCA and posterior ICA SWV were significantly higher in the irradiated side. A radiation dose–effect was only (weakly) apparent for PWV (R^2^: end-systolic = 0.067, begin-systolic = 0.155). Ultrasound measurements had good–excellent intra- and interobserver reproducibility. Plaques had similar characteristics but were more diffuse in the irradiated side.

**Conclusions:**

Increased CCA-IMT and SWV in some segments were seen in irradiated carotids. These alterations, even in young patients, mark the need for surveillance of radiation-induced vasculopathy.

*Trial registration*: clinicaltrials.gov (https://clinicaltrials.gov/ct2/show/NCT04257968).

## Background

Better diagnostic and treatment regimens have increased head and neck cancer (HNC) survival [1]. With more survivors, attention to long-term complications of treatment is important. Cardiovascular diseases (CVD) are the leading non-malignant cause of death in HNC survivors [2]. The risk of ischemic cerebrovascular events is minimally doubled in HNC survivors treated with neck irradiation compared to the general population [3].

Although radiation is known to cause microvascular damage, long-term effects on large vessels are less well studied. Intima-media thickness (IMT) is a widely-used, validated measure of atherosclerotic disease associated with cerebrovascular events [4]. Longitudinal studies showed higher IMTs in HNC patients > 5 years after neck irradiation [3, 5]. However, the exact pathophysiology of radiation-induced carotid vasculopathy is unknown. It is unclear whether it differs from atherosclerosis associated with traditional cardiovascular risk factors (CVRFs), such as hypertension and smoking [4]. As most studies compare HNC survivors with healthy volunteers, correction for these confounding factors is needed to assess unbiased radiation effects.

Vascular stiffness might be an early marker of radiation-induced vascular injury and correlates with cardiovascular events [4]. The hypothesis is that radiation of the arterial wall results in occlusion of the vasa vasorum. This leads to loss of elastic tissue and muscle fibers that are replaced with fibrotic, i.e. stiffer, tissue [3]. Innovative, non-invasive ultrasound techniques assessing tissue stiffness could thus aid to determine radiation-induced vasculopathy. These techniques include shear wave elastography (SWE) [6] and pulse wave velocity (PWV) estimation [7]. In SWE, an acoustic radiation force impulse is used to induce shear waves that propagate perpendicular to the ultrasound beam. The shear wave velocity (SWV) is directly related to the tissues’ elasticity. The higher the SWV, the stiffer the tissue [6]. The PWV is the velocity at which pressure waves, generated by the systolic heart contraction, propagate along the arterial tree [7]. Higher PWVs correlate with stiffer arteries. Originally, the PW is tracked from the femoral to the carotid artery to assess aortic stiffness. Nowadays, it can be tracked locally in the carotid artery. As this method determines the regional stiffness, it is better suited to detect radiation-induced carotid vasculopathy.

This study aimed to assess radiation-induced carotid vasculopathy in a unique patient cohort ≥ 5 years after unilateral neck irradiation. Vasculopathy was determined by carotid wall thickness and stiffness, quantified in terms of IMT and SWV and PWV. To minimize concomitant effects of traditional CVRFs, the non-irradiated carotid served as internal control. Plaques were further characterized using magnetic resonance imaging (MRI). Additionally, the relation between radiation dose and ultrasound parameters was explored.

## Methods

This study aimed to determine long-term vascular complications of neck irradiation in young to middle-aged adults. Patients treated between 2010 and 2015 were identified via the radiotherapy database at the Radboud university medical center. Patients were eligible when diagnosed with a head and neck neoplasm between the age of 18 and 40 years and treated with unilateral irradiation ≥ 5 years before inclusion. Exclusion criteria were contraindications to MRI or insufficient command of Dutch. This study was approved by the local Medical Ethics Review Committee and conformed to the principles of the Declaration of Helsinki. It was registered at clinicaltrials.gov (NCT04257968). All subjects provided written informed consent.

### Patient characteristics

Patient demographics and treatment history were assessed. CVRFs were determined based on the European guidelines on CVD prevention [8], including (I) *Smoking*: current/former with pack-years; (II) *Family history of CVD*: first-degree male ≤ 55 years and/or female ≤ 65 years with CVD; (III) *Hypertension*: systolic blood pressure > 140 mmHg and/or antihypertensive drug use; (IV) *Diabetes mellitus*: non-fasting serum glucose > 11.1 mmol/L and/or antidiabetic drug use; (V) *Hypercholesterolemia*: serum low-density lipoprotein ≥ 2.6 mmol/L and/or non-high-density lipoprotein ≥ 3.4 mmol/L; (VI) *Overweight*: body mass index ≥ 25 and/or abdominal circumference women ≥ 88 cm/men ≥ 102 cm; (VII) *Chronic daily stress*: daily stress > 6 months (work/private); and (VIII) *Chronic renal insufficiency*: estimated glomerular filtration rate < 90 ml/min/1.73 m^2^ and/or albumin-to-creatinine ratio > 3.

### Radiation therapy and dose assessment

All patients were treated with external beam radiotherapy using a linear accelerator (6-MV photon beams) with a three-dimensional conformal or intensity-modulated radiotherapy technique. Total doses were 30–100 Gy in 2-Gy fractions. Radiotherapy targets were defined by computed tomography (CT) and included the primary tumor side with/without the ipsilateral neck. The radiotherapy planning-CT scans were used to determine radiation doses on the carotids. The carotids were delineated using Pinnacle treatment planning software (Version 16.0, Philips Radiation Oncology Systems, Fitchburg, WI, USA) and divided into four segments. The communal carotid artery (CCA) was evenly divided into the proximal, middle, and distal CCA. The fourth segment was the part of the internal carotid artery (ICA) that could be visualized during ultrasound. Mean doses were automatically calculated by the Pinnacle software.

### Ultrasound measurements

Bilateral carotid ultrasounds were performed using an Aixplorer Ultimate system (Hologic Supersonic, Aix-en-Provence, France) with SL18-5 and SL10-2 probes. Blood pressure was measured before and after the ultrasound to correct for blood pressure-dependent stiffness alterations using linear regression. Three B-mode and SWE-mode cineloops lasting three cardiac cycles and ten seconds (≈10 SW-frames), respectively, were acquired in all carotid segments and stored for offline analysis. Three PWV-mode acquisitions were performed. Optimized SWE-acquisition settings included: acoustic power = maximum; smoothing = 6; persistence = off; gain = 65–70%; SWE option = penetration; scale = 0–180 kPa. Patients were asked to hold their breath and avoid swallowing during SWE and PWV acquisitions.

Spatial-averaged IMTs were assessed on the B-mode cineloops in the distal CCA and ICA using in-built automated edge-detection software. Following the Mannheim criteria [9], CCA-IMT was assessed two centimeters proximal to the carotid bifurcation and ICA-IMT ≥ 5–10 mm distal to the bifurcation; both over a segment of 10–15 mm in the posterior wall. Plaques were defined as an IMT ≥ 1.5 mm [9]. Begin- and end-systolic PWV were assessed in the mid-CCA using automated software of the Aixplorer system. Only measurements with a standard deviation < 1 m/s were included. SWV estimations in all carotid segments were performed on the SWE-cineloops using a home-built Matlab (MathWorks, Massachusetts, USA) analysis tool. Region-of-interests (ROI) were manually drawn in the anterior or posterior arterial wall of the first SW-frame to assess anterior or posterior SWVs, respectively. As substantially higher values were seen at the lateral image borders, ROIs were defined ≥ 5 mm from the sides. The ROI was projected on all SW-frames and the mean SWV was calculated for every frame. Mean SWVs of all acquired SW-frames were averaged to obtain a representative SWV estimate, unaffected by the timing of a single SWV estimation, because SWV varies throughout the cardiac cycle [10].

To improve the reliability, all acquisitions including analysis were performed three times and the average was taken as the final measure. To assess the inter- and intra-observer reproducibility of IMT and SWV analyses, analyses were performed by two observers and later repeated by one. Both readers were blinded to clinical data and each other during analysis.

### MRI neck

Patients with a plaque during carotid ultrasound (IMT ≥ 1.5 mm) underwent a neck MRI for plaque characterization. A 3.0 Tesla MR-scanner (Skyra, Siemens Erlangen) with a 3 T-TIM neck coil (Siemens AG, Head Neck 20, Munich, Germany) was used. The MRI protocol consisted of transversal T1-weighted spin-echo (T1-SE) and T2-weighted turbo spin-echo (T2-TSE) sequences, a coronal T1-TSE, and a three-dimensional time of flight (3D-TOF) angiography from the thoracic outlet to the skull base. Scan parameters are stated in Table 5 (“Appendix 1”).

Two observers evaluated the MR-scans for image quality and plaque characteristics bilaterally in the CCA and ICA. Image quality was assessed on a four-point scale (1 = unusable, 4 = optimal) per image sequence (i.e. T1, T2, and 3D-TOF). Images with a quality score of one were excluded. First, the visibility of wall thickening on the MR-images was evaluated as the resolution of MRI is lower compared to ultrasound. Plaques were evaluated on thickness (mm), length (mm), distance from the carotid bifurcation (mm), circumferential extension (0°–90°/90°–180°/180°–270°/270°–360°), cap disruption (yes/no), ulceration (yes/no), stenosis degree (0%/0–50%/50–99%/occlusion [11]), and signal intensity per image sequence compared to the sternocleidomastoid muscle (hypo-/iso-/hyperintense). Plaque thickness, length, and distance from the bifurcation were averaged over both observers. Other results were compared, and consensus was obtained in case of inconsistencies.

### Statistical analysis

Given the small sample size, data were expressed in medians with interquartile ranges and statistical analysis was performed using non-parametric tests. Differences in ultrasound measurements between irradiated and control sides were visualized in dot-/boxplots and statistically tested using Wilcoxon signed-rank tests. The relation between radiation dose and ultrasound parameters was explored with scatter plots and (multivariate) linear regression. A correction was performed for possible factors influencing wall thickness and stiffness, i.e. age, CVRFs, systolic blood pressure, and concomitant chemotherapy [12, 13]. We did not correct for multiple testing due to the explorative study design and relatively small sample size. Intra-observer reproducibility of ultrasound acquisitions including analysis was expressed by the intraclass correlation coefficient (ICC) of three consecutive acquisitions. The intra- and interobserver reproducibility of IMT and SWV analyses were quantified as the ICC between two observers and within one observer, respectively. MR measurements were summarized and qualitatively compared between irradiated and control carotids.

## Results

**Fig. 1:**
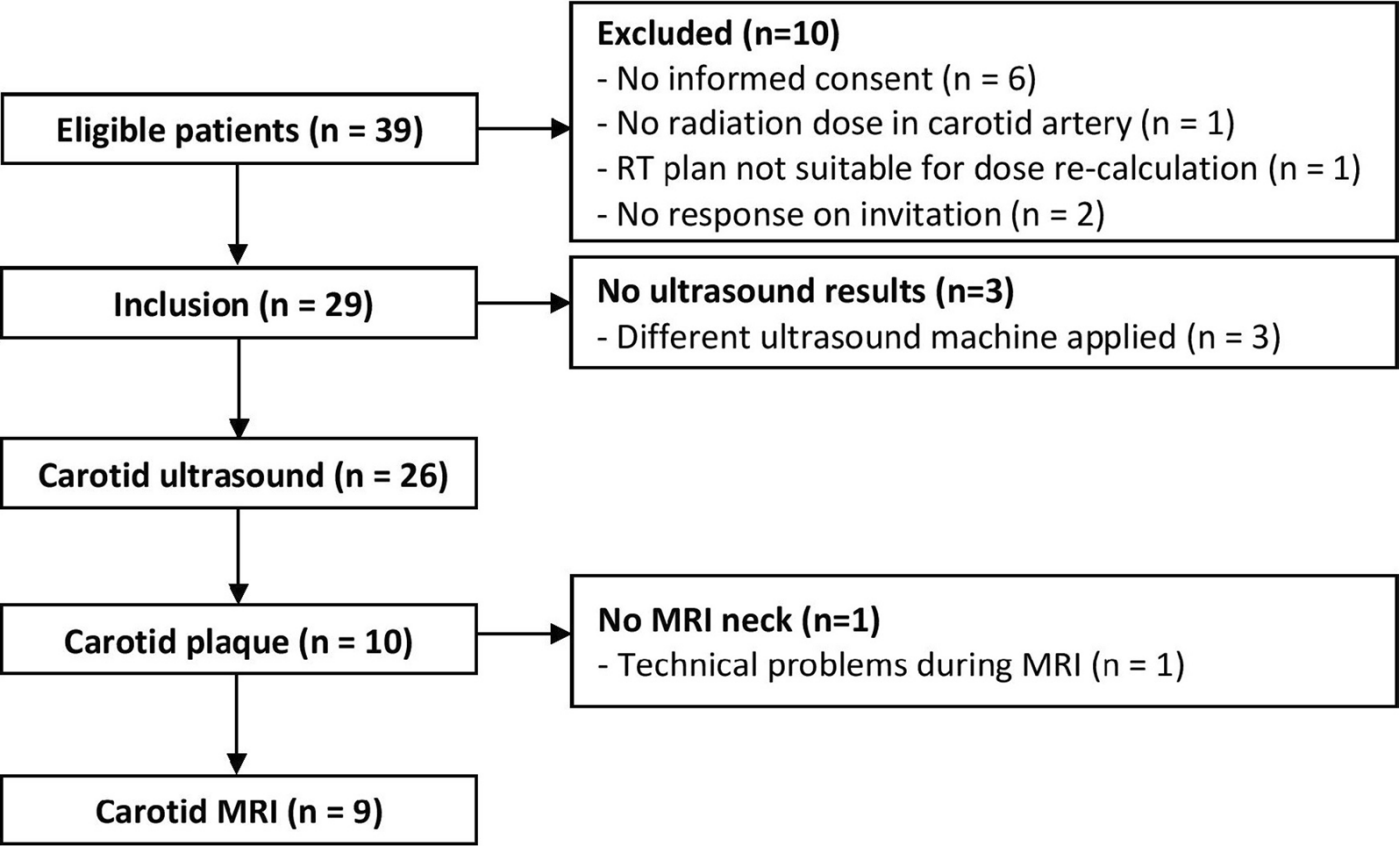
Flowchart of patient inclusion. *NPA* neuropsychological assessment, *RT* radiotherapy, *MRI* magnetic resonance imaging

Twenty-nine patients were included. A flowchart of patient inclusion is shown in Fig. 1*.* Thirteen patients were excluded due to reasons stated in Fig. 1. In three patients, plaque screening was performed with a different ultrasound system, i.e. Mindray DC80A (Mindray Medical, Shenzhen, China) with an L14-5WE transducer due to temporary technical problems with the Aixplorer. Further ultrasound measurements were not performed in these patients because of differences in transducer characteristics and analysis software. Ten patients had carotid artery plaque(s) during ultrasound examination, of which nine underwent a neck MRI.

### Patient characteristics

Patient characteristics are shown in Table 1. Median age at inclusion was 40.5 [33.8–44.8] years. Median follow-up after radiotherapy was 7.2 [6.0–10.0] years. Eighty-eight percent of patients had one or more CVRFs including mostly hypercholesterolemia, overweight, and chronic daily stress. Cholesterol levels and renal function were unknown in five and two patients, respectively. As most patients had parotid tumors, highest radiation doses were located in the distal CCA and ICA.

**Table 1:** Patient characteristics

Characteristic	Patients scanned with Aixplorer system (n = 26)
***Demographics***	
Men, n (%)	12 (46)
Median age, years [IQR]	40.5 [33.8–44.8]
Median follow-up after RT, years [IQR]	7.2 [6.0–10.0]
***Diagnosis, n (%)***	
Carcinoma of parotid	6 (23)
Pleiomorphic adenoma of parotid	8 (31)
Carcinoma of oropharynx	2 (8)
Malignant lymphoma	7 (27)
Others	3 (12)
***Chemotherapy, n (%)***	
Anthracycline	7 (26)
Platinum-based	1 (4)
Alkylating	7 (26)
Other	1 (4)
Total	8 (31)
***Surgery***	
(Partial) parotidectomy	15 (58)
Cervical lymph node dissection	5 (19)
Partial glossectomy	2 (8)
(Hemi)mandibulectomy	2 (8)
Other	2 (8)
Total	18 (69)
***CVD risk factors, n (%)***	
Smoking	
- No	17 (65)
- Current	4 (15)
- Former	5 (19)
- Pack years, median [IQR]	16.0 (9.5–27.0)
Positive family history of CVD	7 (27)
Chronic daily stress	8 (31)
Hypertension	7 (27)
Hypercholesterolemia	8 (38)
Overweight	9 (39)
Diabetes	1 (4)
Renal insufficiency	3 (13)
***Number of CVD risk factors, n (%)***	
0	3 (12)
1	6 (23)
2	10 (39)
≥ 3	7 (27)
***Radiation dose, Gy (median [IQR], min–max)***	
Proximal CCA^2^	1 [0–31], 0–55^3^
Mid-CCA^2^	4 [1–30], 1–55^3^
Distal CCA^2^	22 [8–39], 1–55
ICA^2^	43 [29–52], 1–59
Total applied to the neck	50 [38–66], 30–100^1^

*RT* radiotherapy, *CVD* cardiovascular disease, *CCA* common carotid artery, *ICA* internal carotid artery

^1^One patient underwent a re-irradiation for recurrence of the primary tumor resulting in a total dose of 100 Gy

^2^Carotid on irradiated side

^3^Patients with parotid gland tumors generally received low radiation doses in more proximal common carotid artery segments, but higher doses in the distal common carotid artery and internal carotid artery

### Carotid ultrasound

**Fig. 2:**
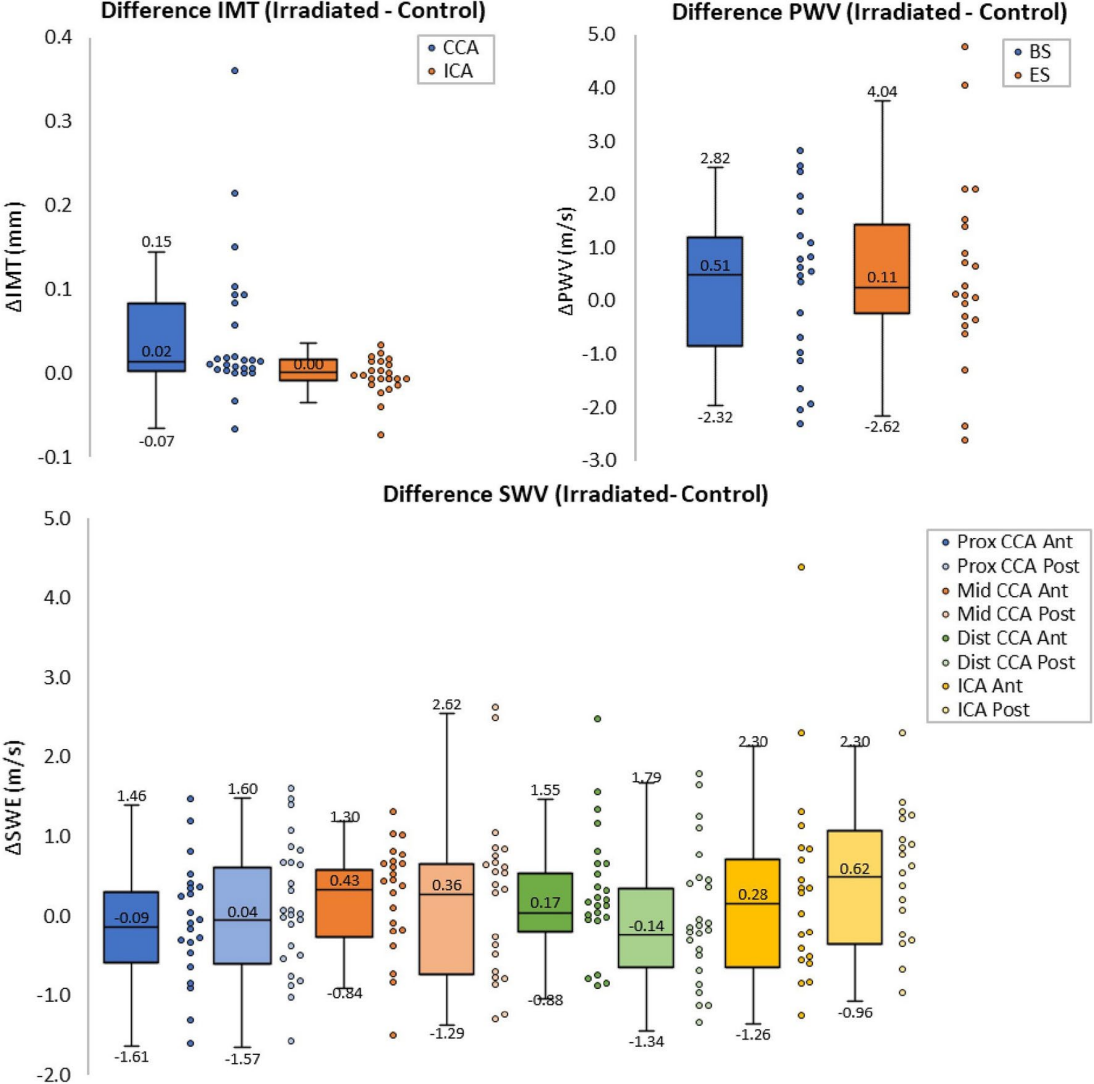
Box- and dot plots of differences in intima-media thickness (upper left), pulse wave velocity (upper right), and shear wave velocity (bottom) between the irradiated and control side in different carotid segments. *IMT* intima-media thickness, *CCA* common carotid artery, *ICA* internal carotid artery, *PWV* pulse wave velocity, *BS* begin-systolic, *ES* end-systolic, *SWV* shear wave velocity, *Prox *proximal, *Dist *distal, *Ant* anterior, *Post* posterior. *Significant difference (p < 0.05) between irradiated and control side

Obtained ultrasound measurements are listed in Table 2. Eight patients undergoing the entire ultrasound protocol had carotid plaque(s): five in the irradiated side, one in the control side, and two bilaterally. Of the three patients screened with the Mindray, one had a plaque in the control side and one bilaterally. Box/dot plots of differences between the irradiated and control side are shown in Fig. 2. CCA-IMT, but not ICA-IMT, was significantly higher in irradiated carotids. Although overall stiffness seemed higher in the irradiated side, stiffness differences were highly variable. Only anterior mid-CCA and posterior ICA SWVs were significantly higher in the irradiated side.

**Table 2:** Measured ultrasound parameters irradiated and non-irradiated side

Parameter	Location	Time point	Irradiated side	n	Control side	n	P value
Plaque	CCA/ICA	n/a	7	26	3	26	0.29^1^
IMT (mm)	Distal CCA	Diastole	0.54 [0.50–0.61]	26	0.50 [0.44–0.54]	26	0.001*
	ICA		0.45 [0.44–0.46]	26	0.45 [0.44–0.47]	23	0.59
PWV (m/s)	Mid-CCA	Begin-systole	4.89 [4.22–5.75]	25	4.56 [3.78–5.58]	22	0.36
		End-systole	6.77 [5.82–7.78]	24	6.44 [5.16–6.97]	22	0.24
SWV (m/s)	Proximal CCA	Averaged over cardiac cycle	A: 4.13 [3.56–4.61]	26	A: 4.17 [3.91–4.39]	25	0.48
			P: 4.30 [3.88–4.71]	26	P: 4.06 [3.84–4.57]	26	0.42
	Mid-CCA		A: 4.68 [4.14–5.04]	26	A: 4.44 [3.63–4.70]	24	0.03*
			P: 4.52 [4.08–5.34]	26	P: 4.50 [4.05–5.03]	24	0.46
	Distal CCA		A: 4.67 [3.75–5.10]	25	A: 4.25 [3.58–4.61]	26	0.06
			P: 4.40 [3.96–5.24]	25	P: 4.48 [3.84–5.13]	26	0.74
	ICA		A: 2.63 [2.29–3.54]	23	A: 2.67 [2.33–3.18]	22	0.34
			P: 2.84 [2.34–3.19]	22	P: 2.23 [1.82–2.62]	19	0.01*

*IMT* intima-media thickness, *PWV* pulse wave velocity, *SWV* shear wave velocity, *CCA *common carotid artery, *ICA *internal carotid artery, *A* anterior; *P* posterior

^*^Significant difference (p < 0.05) between irradiated and control side

^1^Fisher’s exact test

### Radiation dose-effects

**Fig. 3:**
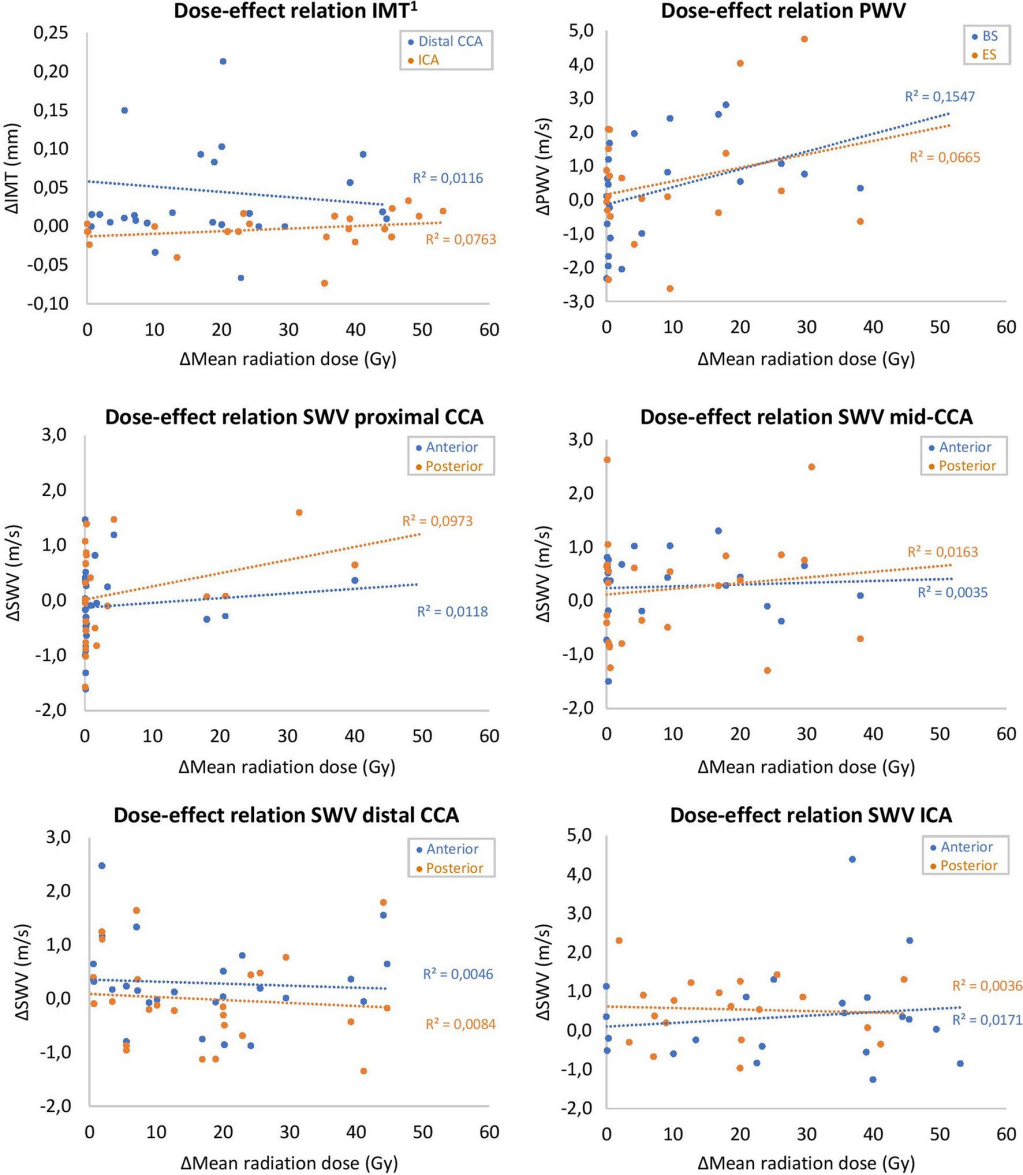
Scatterplots of radiation dose–effect relation for intima-media thickness (upper left), pulse wave velocity (upper right), and shear wave velocity in the different carotid segments (middle left to lower right). Differences in ultrasound parameters are plotted against differences in radiotherapy dose in the irradiated compared to the control side. *IMT* intima-media thickness, *PWV* pulse wave velocity, *SWV* shear wave velocity, *BS* begin-systolic, *ES* end-systolic, *CCA* common carotid artery, *ICA* internal carotid artery. ^1^One outlier is not shown with an IMT and dose difference of 0.36 mm and 1.9 Gy, respectively

Scatterplots of dose–effect relations are shown in Fig. 3. A radiation dose–effect relation only seemed apparent, although weak, for PWV (R^2^: end-systolic = 0.067, begin-systolic = 0.155). Regression coefficients of dose–effect relations are stated in Table 6 (“Appendix 2”). None were statistically significant and correction for possible confounders did not substantially affect coefficients.

### Reproducibility of ultrasound measurements

Intra-observer reproducibility of acquisitions including analysis was excellent for CCA-IMT but worse for ICA-IMT (ICC = 0.972 vs. 0.657), and somewhat higher for SWV in most segments than for PWV (ICC = 0.745–0.919 vs. ICC = 0.707–0.725) (Table 3). Intra- and interobserver reproducibility of analysis were good for CCA-IMT but low for ICA-IMT (ICC = 0.930 vs. 0.570 and ICC = 0.939 vs. 0.272, respectively), and excellent for SWV (ICC = 0.974–0.998 and ICC = 0.963–0.996). No differences between segments or arterial walls were observed.

**Table 3:** Plaque characteristics on magnetic resonance imaging in the irradiated and control side

Side	Patient	Age (yr)	Sex	F/u (yr)	Plaque	Thickness (mm)	Length (mm)	Dist. (mm)	Circ. (°)	Ulceration	Disruption	Intensity 3DTOF/T1/T2^1^	Stenosis (°)
Irradiated side	1	49	M	4.3	–	–	–	–	–	–	–	–	–
	2	61	M	10.0	ICA	3.1	20	0	0–90	No	No	Iso/iso/hyper	0–50
					ICA	2.5	19	13	180–270	No	No	Hypo/hyper/hyper	0–50
	3	44	M	6.5	ICA	2.5	14	0	0–90	No	No	Iso/iso/hyper	0
	4	42	F	5.9	CCA	2.7	12	0	90–180	No	No	Hypo/hyper/hyper	0–50
					ICA	2.0	11	0	90–180	No	No	Hypo/hyper/hyper	0–50
	5	42	F	8.7	CCA	3.0	29	0	270–360	No	No	Iso/iso/hyper	0–50
	6	34	F	12.6	–	–	–	–	–	–	–	–	–
	7	55	F	13.1	ICA	4.1	28	0	180–270	No	No	Hypo/iso/hyper	0–50
	8	51	F	12.9	CCA	2.4	16	0	270–360	No	No	Iso/iso/hyper	0
Control side	1	49	M	4.3	ICA	2.5	12	0	0–90	No	No	Hypo/iso/hyper	0
	2	61	M	10.0	ICA	2.8	7	0	90–180	No	No	Iso/hyper/iso	0–50
	3	44	M	6.5	–	–	–	–	–	–	–	–	–
	4	42	F	5.9	–	–	–	–	–	–	–	–	–
	5	42	F	8.7	–	–	–	–	–	–	–	–	–
	6	34	F	12.6	–	–	–	–	–	–	–	–	–
	7	55	F	13.1	–	–	–	–	–	–	–	–	–
	8	51	F	12.9	CCA	2.9	12	0	0–90	No	No	Iso/iso/hyper	0

*F/u* follow-up, *Dist.* distance from carotid bifurcation, *Circ* circumferential expansion, *3D-TOF* 3-dimensional time of flight angiography, *M* male, *F* female, *ICA* internal carotid artery, *CCA* common carotid artery

^1^As compared to the adjacent sternocleidomastoid muscle

### MRI neck

MR-based plaque characteristics are shown in Table 4. Eleven plaques were observed; eight in irradiated and three in control carotids. Due to the lower resolution of MRI, plaques observed during ultrasound in two patients were not seen during MR-evaluation. Although most plaques were located in irradiated carotids, they were small (stenosis < 50%) and had no signs of instability, i.e. surface ulcerations or cap disruption. Plaque intensities were comparable in both sides. However, plaques were more diffuse in irradiated than control carotids: circumferential expansion ≤ 360° versus ≤ 90°–180° and length 11–28 mm versus 7–12 mm. Although a limited number of plaques was observed, patients with plaque(s) generally had a longer follow-up and more CVRFs than patients without plaque(s) (9.3 [6.5–12.8] vs. 7.6 [6.8–6.2] years and 2.5 [2.0–3.0] vs. 2 [1.0–2.0], respectively). Radiation doses were similar in both groups.

**Table 4:** Intra- and interobserver reproducibility of the entire process (acquisition including analysis) and of solely the analysis of ultrasound measurements

Parameter	Location	Time point	Process (n = 26)	Analysis (n = 10)	
			Intra-observer ICC (95% CI)	Intra-observer ICC (95% CI)	Inter-observer ICC (95% CI)
IMT (mm)	Distal CCA	Diastole	0.972 (0.956–0.983)	0.930 (0.833–0.972)	0.939 (0.853–0.976)
	ICA		0.657 (0.510–0.778)	0.570 (0.167–0.726)	0.272 (− 0.100–0.607)
PWV (m/s)	Mid-CCA	Begin-systole	0.725 (0.554–0.852)	n/a^1^	n/a^1^
		End-systole	0.707 (0.551–0.828)	n/a^1^	n/a^1^
SWV (m/s)	Proximal CCA	Averaged	A: 0.873 (0.804–0.922)	A: 0.998 (0.994–0.999)	A: 0.991 (0.977–0.997)
			P: 0.745 (0.628–0.837)	P: 0.995 (0.979–0.998)	P: 0.978 (0.867–0.994)
	Mid-CCA		A: 0.919 (0.874–0.950)	A: 0.997 (0.992–0.999)	A: 0.995 (0.986–0.998)
			P: 0.876 (0.811–0.923)	P: 0.997 (0.984–0.999)	P: 0.986 (0.885–0.996)
	Distal CCA		A: 0.899 (0.846–0.937)	A: 0.997 (0.992–0.999)	A: 0.996 (0.989–0.998)
			P: 0.881 (0.820–0.926)	P: 0.998 (0.984–0.999)	P: 0.992 (0.976–0.997)
	ICA		A: 0.916 (0.866–0.950)	A: 0.994 (0.986–0.998)	A: 0.988 (0.970–0.995)
			P: 0.859 (0.773–0.919)	P: 0.974 (0.928–0.990)	P: 0.963 (0.898–0.986)

*IMT* intima-media thickness, *PWV* pulse wave velocity, *SWV* shear wave velocity, *CCA *common carotid artery, *ICA *internal carotid artery, *ICC* intraclass correlation coefficient, *CI* confidence interval, *A* anterior, *P* posterior

^1^Automatic analysis by Aixplorer system so independent of user interpretation

## Discussion

With more, younger HNC survivors, attention to long-term vascular treatment sequelae is required. We studied radiation-induced carotid thickness and stiffness alterations using ultrasound in a unique patient cohort ≥ 5 years after unilateral neck irradiation. CCA-IMT and SWV in some segments were significantly higher in the irradiated than in the control side. A radiation dose–effect relation seemed only apparent for PWV. Ultrasound stiffness-derived parameters had good–excellent intra- and interobserver reproducibility. Plaques were more prevalent and more diffuse in the irradiated side, but they were small and similarly characterized in both sides during MR-evaluation.

CCA-IMT was increased long-term after radiotherapy, independent of CVRF-associated atherosclerosis. Longitudinal studies showed higher IMTs in irradiated than in non-irradiated HNC survivors or healthy controls [3, 5]. Also, as currently found, higher IMTs in irradiated than non-irradiated carotids in unilateral irradiated patients have been described [5, 14, 15]. Because the latter setting eliminates confounding effects of CVRFs, these results point to a specific cause-effect relation between radiation and carotid wall thickening. As previously [5], we did not find a higher ICA-IMT in the irradiated side. This can be related to the lower precision of ICA measurements resulting from the lower reproducibility and lower resolution probe used. Alternatively, radiation toxicity may be more pronounced in the CCA than in the ICA.

A higher stiffness of irradiated than non-irradiated carotids in unilateral irradiated patients was shown with local echo-tracking methods, i.e. elastic modulus (Ep) and beta-stiffness index (β) [17, 16]. We only found slight SWV differences between irradiated and control carotids. Smaller differences in the current study could be explained by lower radiation doses or somewhat shorter follow-ups. Alternatively, accuracies of stiffness estimation techniques could differ. Ep and β are calculated using the intra-arterial blood pressure. This can only be approximated with external blood pressure measurements. Ultrafast imaging methods are independent of blood pressure, possibly providing more accurate stiffness estimations. Besides, a high reproducibility found in this and previous studies [10, 17] point to its user-independence. Studies comparing different stiffness estimation methods are needed to determine optimal techniques for vasculopathy assessment.

So far, radiation dose has been associated with IMT [14], but not with carotid stiffness [12]. Relatively low radiation doses applied in this study could result in the lack of a dose–effect relation with IMT. Martin et al. already described a threshold of 35 Gy for IMT alterations [15]. An, although weak, dose–effect relation for PWV could suggest this method is most sensitive to radiation-induced stiffness alterations. Although we expected a clearer dose–effect relation for SWV due to the more precise, segment-wise evaluation, this was not seen. However, SWV measurements could be suboptimal. Measurements were not electrocardiogram-gated, and push locations and assumed shear wave propagation paths could not be controlled. Further research with higher radiation doses and improved SW-acquisitions is needed to determine segment-wise radiation toxicity.

Lam et al. showed that radiation-induced plaques are more hypoechoic, less calcified, and more often located in the CCA than CVRF-associated plaques [18]. This suggests radiation-induced plaques are less stable. Although we found more plaques in irradiated carotids, they were similarly localized and characterized as in control carotids, consistent with our previous findings [19]. However, more diffuse plaques in irradiated carotids could point to a different pathophysiological mechanism or an acceleration of pre-existing atherosclerotic plaques.

The results of this study must be interpreted considering its limitations. Due to the explorative nature, we included a limited number of patients, and carotid thickness and stiffness data before radiation were unavailable. Moreover, radiation doses were relatively low as patients with benign and malignant neoplasms were included possibly leading to only small vascular alterations. Although our cohort was heterogeneous in diagnosis, treatment regimens always included unilateral irradiation. Large, prospective studies in typical HNC patients (higher age, more CVRFs, higher radiation dose) are indicated to address the prevalence and relevance of radiation-induced carotid vasculopathy in these patients. Additionally, natural differences in thickness [20] and stiffness [17] in left and right carotids have been shown, limiting the principle of an internal control. However, adjustment for the irradiation side did not change our findings.

## Conclusions

We showed the feasibility and high reproducibility of (ultrafast) ultrasound to assess radiation-induced carotid vasculopathy. Carotid thickness and stiffness alterations in irradiated carotids, even in young patients treated with relatively low radiation doses, underline the importance of surveillance for radiation-induced vasculopathy.

## Data Availability

The data that support the findings of this study are available from the corresponding author, JTP, upon reasonable request.

## Appendix 1

See Table 5.

**Table 5:** Magnetic Resonance Imaging scan parameters

Sequence	TR (ms)	TE (ms)	Slice thickness (mm)	Flip angle (°)	Voxel size (mm)
Transversal T1-SE	450	13.0	2.0	70–180	0.3 × 0.3 × 2.0
Coronal T1-TSE	750	24.0	0.9	–	0.4 × 0.4 × 0.9
Transversal T2-TSE	3000	62.0	2.0	180	0.3 × 0.3 × 2.0
3D-TOF	20	3.1	1.0	25	0.5 × 0.5 × 1.0

*TR* repetition time, *TE* echo time, *TSE* turbo spin-echo, *SE* spin-echo, *3D-TOF* three-dimensional time of flight

## Appendix 2

See Table 6.

**Table 6:** Correlation radiation dose and ultrasound parameter differences between irradiated and control side

Parameter	Location	Time point	Regression coeff. RT dose (95% CI)^1^	P	Corrected regression coeff. RT dose (95% CI)^2^	p
ΔIMT (mm)	Distal CCA	Diastole	− 0.001 (− 0.003 to 0.002)	0.60	− 0.001 (− 0.004 to 0.001)	0.35
	ICA		0.000 (0.000 to 0.001)	0.20	0.000 (− 0.001 to 0.001)	0.60
ΔPWV (m/s)	Mid-CCA	Begin-systole	0.040 (− 0.032 to 0.111)	0.26	0.065 (− 0.025 to 0.154)	0.15
		End-systole	0.052 (− 0.005 to 0.110)	0.07	0.048 (− 0.005 to 0.101)	0.07
ΔSWV (m/s)	Proximal CCA	Averaged over cardiac cycle	A: 0.009 (− 0.025 to 0.043)	0.61	0.001 (− 0.038 to 0.040)	0.95
			P: 0.024 (− 0.007 to 0.055)	0.12	0.021 (− 0.017 to 0.060)	0.26
	Mid-CCA		A: 0.003 (− 0.023 to 0.029)	0.79	0.017 (− 0.015 to 0.048)	0.28
			P: 0.011 (− 0.026 to 0.047)	0.55	0.022 (− 0.025 to 0.069)	0.34
	Distal CCA		A: − 0.004 (− 0.028 to 0.020)	0.75	0.005 (− 0.016 to 0.027)	0.60
			P: − 0.006 (− 0.032 to 0.020)	0.66	0.005 (− 0.019 to 0.029)	0.67
	ICA		A: 0.009 (− 0.024 to 0.043)	0.57	− 0.001 (− 0.051 to 0.049)	0.96
			P: 0.001 (− 0.022 to 0.024)	0.93	− 0.007 (− 0.110 to 0.024)	0.63

*RT* radiotherapy, *CI* confidence interval, *IMT* intima-media thickness, *PWV* pulse wave velocity, *SWV* shear wave velocity, *CCA *common carotid artery, *ICA *internal carotid artery, *A* Anterior, *P* posterior

^1^Linear regression coefficient per Gray dose difference

^2^Linear regression coefficient per Gray dose difference corrected for age, systolic blood pressure during ultrasound examination, number of cardiovascular risk factors, and concomitant chemotherapy

## Abbreviations

CCA: Common carotid artery
CT: Computed tomography
CVD: Cardiovascular diseases
CVRF: Cardiovascular risk factors
HNC: Head and neck cancer
IMT: Intima-media thickness
ICA: Internal carotid artery
ICC: Intraclass correlation coefficient
MRI: Magnetic resonance imaging
PWV: Pulse wave velocity
ROI: Region-of-interest
SWE: Shear wave elastography
SWV: Shear wave velocity
